# An online gene expression assay for determining adjuvant therapy eligibility in patients with stage 2 or 3 colon cancer

**DOI:** 10.1038/sj.bjc.6605970

**Published:** 2010-11-30

**Authors:** R K Van Laar

**Affiliations:** 1 ChipDX LLC, PO Box 286874, New York, NY 10128, USA

**Keywords:** colon cancer, gene expression, prognosis, personalised medicine

## Abstract

**Background:**

The decision whether to treat patients with non-metastatic colon cancer with adjuvant chemotherapy is determined by clinical staging, frequently resulting in over or undertreatment.

**Methods:**

Gene expression data and clinical information from 232 stage 1–4 colon cancer patients were analysed to identify expression patterns predictive of recurrence. The signature was evaluated on an independent series of 60 stage 2 and 3 patients. Multivariate analyses were performed to assess the clinical utility of the assay.

**Results:**

A 163-probe signature was able to stratify patients into high- and low-risk groups for disease-free survival (DFS) in both the training and validation series (stage 2: *P*⩽0.031, stage 3: *P*⩽0.057) and for disease-specific survival in the training series (stage 2: *P*=0.01, stage 3: *P*=0.0017). Multivariate analysis showed the classifier to be associated with approximately three- to fourfold increased risk of recurrence.

**Conclusions:**

The prognostic gene expression signature is able to stratify stage 2 and 3 colon cancer patients into groups with significant differences in 5-year DFS, information that may ultimately reduce deaths from colon cancer. Further validation work is required and at this stage the assay is available for evaluation at www.ChipDX.com.

Each year, tumours of the colon are responsible for 655 000 deaths globally (2009). In the USA, it is the fifth most common type of cancer, with an estimated 106 100 new cases annually (Jemal *et al*, 2008). Fortunately, most patients are diagnosed in the early stages of the disease (i.e., stage 1 or 2), when surgical resection is usually curative. If the cancer has spread to regional lymph nodes (stage 3), surgery and adjuvant chemotherapy (ACTx) represent the current standard of care (Obrand and Gordon, 1997; Markowitz and Bertagnolli, 2009).

The use of staging as the primary means of determining ACTx eligibility results in undertreatment of some stage 2 patients, who generally do not receive chemotherapy, but experience disease recurrence in approximately 20% of cases (Quasar Collaborative *et al*, 2007). Improved methods for identifying stage 2 patients at high risk of recurrence, based on the unique characteristics of each individual tumour may result in thousands of lives saved each year. Conversely, studies have shown that practise of treating all stage 3 patients with ACTx results in overtreatment of a substantial portion of cases (Sargent *et al*, 2009). Although chemotherapy has been shown to improve survival for patients with stage 3 cancers, surgery alone is curative for 43% of individuals in this group. Techniques for identifying those stage 3 patients who are at low risk of disease recurrence after surgery may spare these individuals from the cost, time and toxicity associated with chemotherapy.

The use of gene expression profiling to develop new tools for identifying patients at high risk of disease recurrence has been explored by a number of groups. Smith *et al* (2009) refined a metastasis-associated gene expression profile, originally identified in a mouse model of colorectal cancer, to a 34-gene signature associated with metastasis and death in human patients. In stage 3 patients, the hazard ratio for recurrence was 4.7 (95% CI: 1.57–14.05). Jorissen *et al* (2009) used the gene expression difference between stage 1 and stage 4 tumours to create a 128-gene classifier that stratified stage 2 and stage 3 patients into groups with significant differences in outcome. The treatment-adjusted hazard ratio of their classifier, when applied to stage 3 patients, was 2.9 (95% CI: 1.1–7.6), although a result was not generated for 16% of all patients tested, limiting the clinical utility of this assay.

In the present study, gene expression data from stage 1–4 colon cancer patients (Jorissen *et al*, 2009) were analysed using Cox survival models to identify genes associated with outcome, over and above the level of traditional prognostic factors such as age, grade and staging (Cox, 1972). A predictive algorithm was trained using all genes identified by this selection process and evaluated on an independent series of 60 stage 2 and 3 colon cancer patients (Smith *et al*, 2009).

The algorithm has been implemented into the ChipDX online analysis system (http://www.ChipDX.com), compatible with the widely available Affymetrix GeneChip platform. The prognostic signature and online analysis system may allow clinicians to incorporate gene expression profiling into the management of colon cancer patients, without the time, logistics and expense involved in sending biopsy material to an external reference laboratory. Analysis of genomic data using the ChipDX Colon Cancer Module is currently free for evaluation purposes and non-diagnostic use.

## Patients and methods

### Patients and gene expression data

A database of clinical and gene expression data was compiled from a previously described patient series (Smith *et al*, 2009) to identify individual genes with expression patterns significantly associated with prognosis and train an algorithm to predict colon cancer recurrence. This database was comprised of 232 whole-genome Affymetrix U133 Plus 2.0 profiles, generated from fresh-frozen biopsies from colon cancer patients diagnosed with stage 1–4 disease (NCBI GEO: GSE17538). These patients were treated at either the Vanderbilt Medical Centre (Nashville, TN, USA) or the H Lee Moffitt Cancer Center (Tampa, FL, USA) and are described in detail in the original publication. Data were available for age at diagnosis, gender, tumour grade, AJCC stage, and disease-free survival (DFS) and disease-specific survival (DSS).

To objectively assess the significance of the prognostic algorithm developed, an independent validation series of 60 Affymetrix U133 Plus 2.0 profiles from stage 2 and 3 colon cancer patients from another previously published study was used (Jorissen *et al*, 2009). This clinical validation series (GEO ID: GSE14333) was generated from tumour biopsies obtained from consecutive colon cancer patients treated at Westmead Hospital (Westmead, Australia), The Peter MacCallum Cancer Centre and the Royal Melbourne Hospital (both Melbourne, Australia). All patients were untreated before surgery and data were available for age at diagnosis, gender, tumour grade, AJCC stage and DFS. A summary of training and validation series demographics is shown in Table 1.

As the reproducibility of gene expression data can be influenced by a number of technical factors such as reagent or chip batches and scanning equipment settings, an additional series of Affymetrix U133 Plus 2.0 hybridizations were analysed to assess the stability of the prognostic signature between analysis sites (Bowtell, 1999; Mutter *et al*, 2004). In all, 120 GeneChip CEL files, representing four pools of cell-line RNA, hybridised five times in six different laboratories, were used for this analysis. These data were part of the multi-centre Microarray Quality Control study (MAQC), (GEO ID: GSE5350) (Shi *et al*, 2006).

### Data processing and quality control

All Affymetrix CEL files were processed using MAS5 normalisation and background correction. Probes with low intensity (<100) were excluded and each chip was median centred, based on the expression of the internal 100-probe ‘reference set’, a series of probes selected by Affymetrix based on their low variation between multiple tissue types. Although the authors of the original studies reportedly examined the quality of their hybridizations before analysis, all genomic data were re-analyzed using the ChipDX Quality Module, which was specifically designed for diagnostic applications. This multi-step quality system evaluates factors such as nonspecific background binding, normalisation factors, signal-to-noise ratios and replicate probe variation. GeneChips flagged by the ChipDX Quality Module were excluded from the classifier evaluation analyses. See Supplementary Information for more information.

### Prognostic gene selection and algorithm training

**Figure 1:**
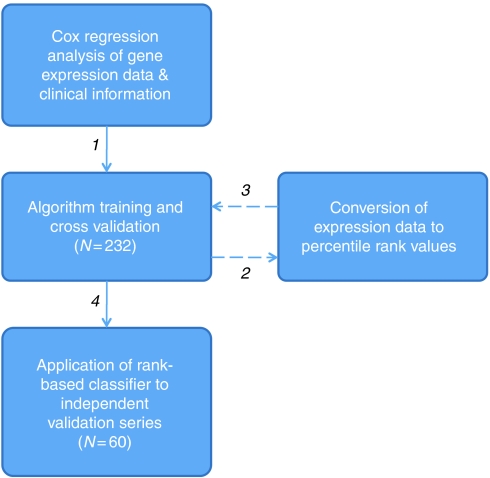
Overview of the analysis performed to develop and validate a novel prognostic gene expression signature for stage 2 and 3 colon cancer. After selection of genes significantly associated with outcome in log-intensity scale, data were converted to percent-rank values and used to re-train the predictive metagene algorithm.

A modified version of the method described by Bair and Tibshirani (2004) was used to develop and train a predictive algorithm capable of stratifying patients into categories corresponding to low or high risk of disease recurrence (Figure 1). This approach uses CPH models to relate survival time to two ‘metagene’ expression levels. These ‘metagenes’ are the first two principle component linear combinations of the corresponding genes found to be significantly associated with recurrence, independent to clinical covariates. The prognostic significance of each gene was assessed using multivariate CPH regression models that included age at diagnosis, tumour grade and clinical staging. In this study, genes with patterns of expression that were significant at *P*<0.002 were used to compute the principle components and regression coefficients (weights).

To apply the classifier on data from a patient whose gene expression profile is described by a vector ‘x’ of log expression levels, the two principle components are computed by combining x with the weights of each linear combination. The weighted average of these two principle component values is then calculated, resulting in a value referred to as the ‘prognostic index’. A high prognostic index corresponds to an increased hazard of colon cancer recurrence. The classification threshold was set based on the 50th percentile of training series indices, which were calculated using leave-one-out cross validation (LOOCV).

After completing this process on the 232-sample training series, expression data for genes selected in 20% or more of the cross-validation rounds were converted to percentile-rank values (range 0.00–100.00) and used to retrain the predictive algorithm. Training-series risk-group predictions from both log-intensity and percentile-rank versions of the algorithm were compared. Finally, the rank-based prognostic algorithm was applied to data from the independent validation series of patients with stage 2 or 3 colon cancer.

### Statistical analysis of risk-group predictions

Kaplan–Meier analysis and log-rank testing was used to evaluate the differences between the predicted risk groups in the training series for 5-year DFS and DSS The independent validation series was evaluated for 5-year DFS only as DSS data was not available. Multivariate Cox proportional hazards (CPH) analysis was performed to determine the independence of the prognostic signature in the presence of clinical covariates. For all tests, *P*-values <0.05 were considered significant.

Gene expression analysis was performed using R (http://www.r-project.org), Bioconductor (Gentleman *et al*, 2004) and BRB ArrayTools (Simon and Lam). Statistical analysis of the prognostic index and risk-group predictions was carried out using MedCalc (MedCalc Inc., Mariakerke, Belgium). A custom R-script was created to encapsulate the diagnostic algorithm and was incorporated into to the ChipDX online analysis system; developed with R, Bioconductor, Microsoft ASP.NET and SQL Server (Microsoft Corporation, WA, USA).

## Results

### Identification of recurrence-associated gene expression patterns

Multivariate analysis of the 232-sample stage 1–4 training series successfully identified a set of 163 probes, significantly associated with colon cancer recurrence, independent to age, grade and stage. An annotated list of the 163 probes and Cox *P*-values is provided in Supplementary Information. The gene set was compared with prognostic colon cancer signatures published by Smith *et al* (34 genes) and Jorissen *et al* (128 genes). No overlap was found between all three signatures, or between the Smith and Jorissen signatures. In all, seven genes were found in common between the Jorissen signature and the 163 probe set identified in this study; AKAP12, DCBLD2, FN1, SPARC, SPP1, THBS2 and VCAN. The hypergeometric probability of this overlap occurring by chance is <1.40 × 10^−7^.

To explore the biological functions of the genes selected to form the prognostic signature, Ingenuity Pathway Analysis software was used (http://www.ingenuity.com). A significant overlap was detected with several relevant gene families, including colon cancer progression (e.g., FN1, IGBP3, PLAUR and TIMP1; *P*=0.00052), tumour cell apoptosis (e.g., BID, TNFRSF21, PHLDA1 and NOTCH1; *P*=1.46 × 10^–6^) and cell proliferation (e.g., CTGF, SPP1, FOLR1 and SPARC). Enrichment of genes from the IGF-1 signalling and VDR/RXR activation canonical pathways (*P*=7.82 × 10^−4^ and *P*=3.85 × 10^−3^ respectively) was also found. These molecular pathways have been implicated in colon cancer development and progression (Khandwala *et al*, 2000; Wactawski-Wende *et al*, 2006).

**Figure 2:**
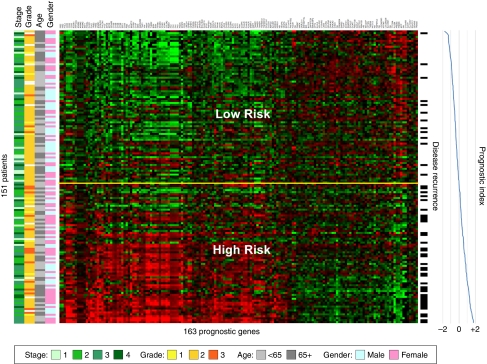
Gene expression heatmap of the 163-probe colon cancer prognosis signature in a subset of the stage 1–4 training series. Patients without recurrence and less than 3-years follow-up, or recurrence events >10 years, have been excluded. Rows indicate patients (ordered by prognostic index), columns indicate probes (ordered by hierarchically clustering using average linkage similarity). Red colour indicates high gene expression, green colour indicates low gene expression (log2 scale). Recurrence events are indicated to the right of the heatmap. Yellow horizontal line corresponds to the classification threshold, determined during algorithm training. The colour reproduction of this figure is available on the html full text version of the manuscript.

A gene expression ‘heatmap’ of the 163-probe signature in the training series is shown in Figure 2, in which genes (columns) are arranged by hierarchical clustering and patients (rows) are ordered according to their prognostic index. The relationship between gene expression and disease recurrence can be observed in the pattern of upregulation and downregulation formed by this arrangement, with higher expression of those genes on the left of the heatmap associated with poor prognosis and vice versa. An increasing frequency of recurrence events (indicated to the right of the heatmap) can be observed as the prognostic index increases from −2.0 to +2.0.

### Analysis of cross-validated training series risk-group predictions

**Figure 3:**
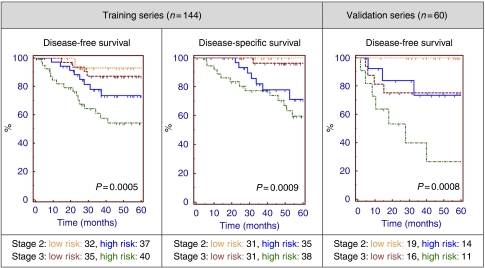
Kaplan–Meier analysis of stage 2 and 3 colon cancer patients stratified by clinical staging and the 163-gene prognostic signature. Risk-group predictions for those patients who were part of the training series (*n*=144) were determined by cross-validation analysis. The final 163-gene classifier was then applied to the independent analysis series (*n*=60) of patients, who were not involved in the gene selection or algorithm development process. *P*-values generated by the log rank test.

In order to reflect the intended use of the assay, risk-group predictions for the subset of the training series with stage 2 or 3 colon cancer (*n*=144) were compared using Kaplan–Meier analysis for DFS and DSS. These predictions were generated using LOOCV, as part of the gene selection and algorithm training process, in order to minimise over fitting of the data (Simon, 2005). Log rank testing revealed a significant difference between the high- and low-risk groups for both DFS (*P*=0.0008, HR: 4.08 95% CI: 1.99–8.34) and DSS (*P*<0.0001, HR 19.59 95% CI: 8.33–46.07), and also for stratification by risk group and clinical staging (Figure 3). For comparison purposes, Kaplan–Meier analysis of these patients stratified by staging was performed, however the result was not statistically significant for either DFS (*P*=0.75) or DSS (*P*=0.30).

No differences in risk-group predictions were observed between versions of the 163-probe algorithm trained on log intensity or percentile-rank expression data. Therefore, the performance of the predictive algorithm was not affected by this data transformation step.

### Analysis of independent clinical validation series

The trained 163-probe algorithm was then applied to data from an independent series of 33 stage 2 and 27 stage 3 colon cancer patients, not involved in the gene selection or algorithm development process. In all, 35 (58%) of these patients were classified as low risk (i.e., prognostic index <50th percentile of cross-validated training series indices; −0.104). Kaplan–Meier analysis and log rank testing of the two risk groups, containing both stage 2 and 3 patients, revealed a significant difference in 5-year DFS (*P*=0.021, HR: 3.19 95% CI: 1.18–8.63), as shown in Figure 3.

Kaplan–Meier analysis of risk groups stratified by gene expression risk group and clinical staging was then performed, resulting in a significant difference in DFS for stage 2 patients (*P*=0.0031) and approaching significance for stage 3 patients (*P*=0.057), as shown in Table 2. Notably, no low-risk stage 2 patient from this series experienced disease recurrence for (up to) 5 years.

### Multivariate analysis of gene expression risk groups

To evaluate the significance of the 163-gene classifier in a multivariate setting, CPH analysis was performed on stage 2 and 3 patients from both training and validation series, including all available clinical covariates (Table 3). For the LOOCV-analyzed training series, this investigation revealed the gene expression-based risk group assignment to be the strongest predictor of both DFS (*P*=0.0018, HR 4.23, 95% CI 1.72–10.39) and DSS (*P*=0.0040, HR: 19.13, 95% CI: 2.59–141.55), independent to age, gender, stage and grade.

CPH analysis of the independent validation series included age, gender, stage, ACTx/ARTx status and gene expression risk group. The gene expression classifier was observed to be a strong predictor of recurrence, with a hazard ratio of 3.04 (95% CI: 0.96–9.69), although it did not achieve independent statistical significance in this model (*P*=0.061). This is possibly because of the limited size of the validation series or the impact of adjuvant therapy on DFS.

### Analysis of replicate hybridizations performed at multiple laboratories

Replicate hybridizations of four RNA pools were analysed to determine the impact interlaboratory variation on the 163-probe prognostic index. In all, five replicate Affymetrix hybridizations of four samples, performed at six separate locations were processed. All 120 samples were classified as ‘high risk’ and no significant difference was observed between the replicate hybridizations performed at any given site (*P*>0.05). Standard deviation of the mean site indices was sample A: 0.032, sample B: 0.051, sample C: 0.029 and sample D: 0.034. The mean s.d. (0.037) represents 1.1% of the prognostic index range observed in the algorithm training series and is used to compute a 95% confidence interval for prognostic index calculations performed in the ChipDX online analysis system.

## Discussion

As the use of chemotherapy for patients with stage 2 and 3 cancer remains controversial (Quasar Collaborative *et al*, 2007), there is a need for improved methods of risk assessment. In this study, multivariate survival models were applied to clinical and gene expression data to identify a prognostic signature for stage 2 and 3 colon cancer. This was used to create a robust diagnostic tool that may ultimately assist clinicians in tailoring personalised treatment options, in conjunction with the clinical staging system.

The ‘meta-gene’ classification algorithm was developed from a multi-centre series of stage 1–4 colon cancer patients and then independently validated on a separate series of stage 2 and 3 colon cancer patients. In the case of patients with stage 2 disease, the assay is able to identify those who are at low risk of disease recurrence; that is, 89% RFS in the training series and 100% RFS in the validation series, for up to 5 years following diagnosis. By comparison, high-risk stage 2 patients experience a 24–27% lower rate RFS, suggesting that adjuvant therapies should be considered for patients assigned to this risk group. Stratification of stage 2 patients also corresponded to a significant difference in DSS in the training series, confirming the clinical significance of the assay.

Patients diagnosed with stage 3 colon cancer are commonly treated with ACTx, yet relapse is still observed in approximately 40% of cases (Andre *et al*, 2004). Genomic stratification of stage 3 patients in this study resulted in groups with significant differences in RFS, with those patients classified as high risk experiencing an extremely poor 5-year RFS rate of 43% (training series) and 26% (validation series). As such, a patient with stage 3 disease and the high-risk gene expression signature may benefit from a more aggressive treatment regimen, possibly including targeted or experimental therapies, such as bevacizumab or panitumumab (Hurwitz *et al*, 2004; Seront *et al*, 2010).

The signature developed in this study differs from previous groups in several ways. First, it was developed exclusively using a training series of gene expression and clinical data derived from human colon tumours, representing all major stages of progression. Tumours of the rectum were intentionally excluded as they are increasingly recognised as a distinct category with a different origins and treatment options (Konishi *et al*, 1999). Each gene in the signature is individually associated with outcome independent to traditional prognostic variables. The algorithm trained on these data uses robust gene expression rank values, rather than log-scale intensities which are more susceptible to inter and intralaboratory technical variation. Finally, the prognostic index is a continuous variable, positively correlated with increased risk of colon cancer recurrence and capable of stratifying patients into risk groups that are statistically and clinically significant, for up to 5 years following diagnosis.

The 163-probe prognostic algorithm is available for evaluation at www.ChipDX.com, which includes an automated GeneChip quality control and result reporting system, designed for the Affymetrix platform. Further validation studies are required to assess the significance of the classifier in additional cohorts of colon cancer patients and to investigate stability on tissues preserved by paraffin fixation or other mediums. Ultimately, the assay developed herein may be used to more accurately select individual patients for potentially life-saving adjuvant therapy, while sparing those who are predicted to have a favourable prognosis.

## Figures and Tables

**Table 1:** Patient demographics of the colon cancer series used for gene selection, algorithm training and independent validation

	**Training series**	**Independent validation series**
*NCBI GEO ID*	*GSE17538*	*GSE14333*
*Contributing institutes*	*Vanderbilt Medical Center (Nashville, TN) & H. Lee Moffit Cancer Center (Tampa, FL, USA)*	*The Peter MacCallum Cancer Centre, Westmead Hospital, & Royal Melbourne Hospital (Australia)*
Number of samples	232	60
Age (years), mean±s.d.	64±13.4	68±13.7
Stage 1, *n* (%)	28 (12%)	—
Stage 2, *n* (%)	72 (31%)	33 (55%)
Stage 3, *n* (%)	76 (33%)	27 (45%)
Stage 4, *n* (%)	56 (24%)	—
Gender: female, *n* (%)	110 (47%)	28 (47%)
Gender: male, *n* (%)	122 (53%)	32 (53%)
Adjuvant chemotherapy	—	22 (37%)
Adjuvant radiotherapy	—	1 (2%)
Median follow-up/survival (months), (range)	30 (0–210)	37 (2–85)
No. recurrences, *n* (%)	55 (23%)	16 (17%)
No. deaths, *n* (%)	93 (40%)	NA

Abbreviation: NA=not applicable.

**Table 2:** Disease-free survival rates for the training and validation series, disease-specific survival rates (maximum) for training series patients (stage 2 and 3) and validation series patients

**Series**	**Clinical Stage**	**Risk group**	**Percent of series**	**5-year DFS (%)**	**Log-rank P-value**	**5-year DSS**	**Log rank P-value**
Training	2	Low	31	89	0.0002	100%	0.010
		High	35	63		70%	
	3	Low	16	87	0.0016	96%	0.0017
		High	18	43		61%	
Validation	2	Low	32	100	0.031		
		High	23	73		NA	
	3	Low	27	75	0.057		
		High	18	26			
							

Abbreviation: NA=not available.

**Table 3:** Multivariate Cox proportional hazards regression of the training & independent validation series (stage 2 and 3 patients) using clinical factors and gene expression risk groups

	**Training series (DFS)**		**Training series (DSS)**		**Validation series (DFS)**	
**Covariate**	**P-value**	**Hazard ratio (95% CI)**	**P-value**	**Hazard ratio (95% CI)**	**P-value**	**Hazard ratio (95% CI)**
Age	0.38	0.99 (0.96–1.01)	0.38	1.01 (0.98–1.05)	0.31	0.98 (0.93–1.02)
Gender (M)	0.59	0.81 (0.38–1.74)	0.97	0.98 (0.40–2.42)	0.22	0.49 (0.16–1.52)
Grade	0.96	1.02 (0.50–2.05)	0.95	1.02 (0.43–2.43)	—	—
Stage (3)	0.07	2.04 (0.95–4.41)	0.28	1.66 (0.65–4.22	0.0021	9.23 (2.26–37.70)
Adjuvant chemotherapy	—	—			0.22	0.43 (0.12–1.62)
Adjuvant radiotherapy	—	—			0.32	3.35 (0.31–36.095)
Gene expression risk group (high risk)	0.0018	4.22 (1.72–10.39)	0.004	19.13 (2.59–141.54)	0.061	3.042 (0.96–9.69)

Abbreviations: CI=confidence interval; DFS=disease-free survival; DSS=disease-specific survival.
