# Supplementary material for: An online gene expression assay for determining adjuvant therapy eligibility in patients with stage 2 or 3 colon cancer
Source: Br J Cancer. 2010 Nov 30;103(12):1852–7. doi: 10.1038/sj.bjc.6605970 (PMC3008603; doi:10.1038/sj.bjc.6605970)
Supplement: Supplementary Information [file 6605970x1.pdf]

An online gene expression assay for determining adjuvant therapy eligibility in patients with stage 2 or 3 colon cancer.

Supplementary Information

Comparison of gene lists used for prediction of colon cancer recurrence:

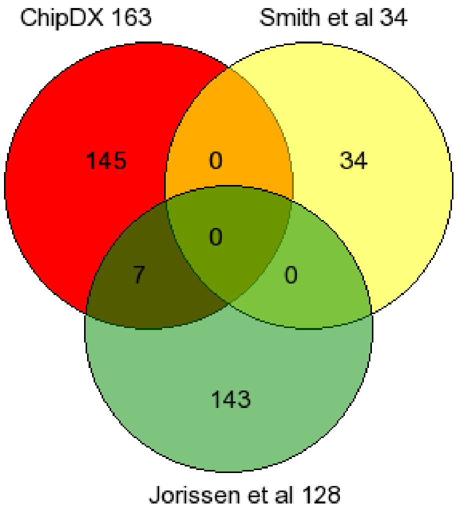

Supplementary Figure 1: Venn diagram of 3 gene expression signatures for colorectal cancer prognosis. 7 genes overlapping between ChipDX and Jorissen et al: AKAP12, DCBLD2, FN1, SPARC, SPP1, THBS2, VCAN.

Analysis of gene expression data quality using the ChipDX Quality Module

The ChipDX 'Quality Control & Normalization Module' is applied to measurements that are generated during chip pre-processing. This is carried out automatically on an individual GeneChip CEL file, prior to any predictive or diagnostic analysis. An easy-to-understand table and series of images are presented to the user which summarizes an extensive array of quality control data and alerts them to any data quality issues which may impact on the accuracy of further analyses.

The ChipDX Quality Module includes multiple assessments of data quality, designed to identify GeneChip hybridizations that are outside of the acceptable 'quality distribution' of samples used to develop the test. The quality assessments made are based on manufacturer recommendations and developer experience with diagnostic gene expression analysis. The metrics chosen are general measurements of microarray quality and not specific to any particular tumor type.

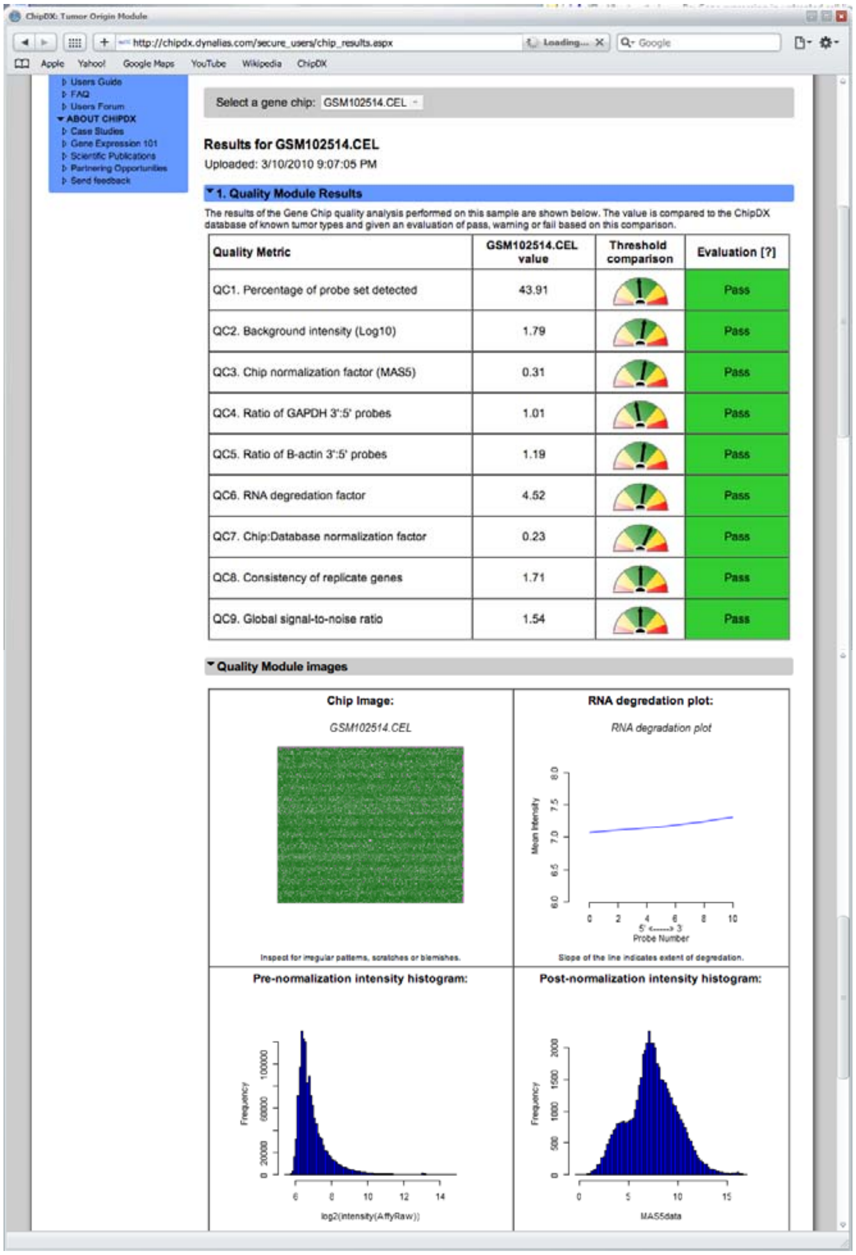

Supplementary Figure 2: Example output from the automated Quality Module, designed to ensure the gene expression data is suitable for diagnostic purposes.

To determine appropriate thresholds for the assessments, quality data were compiled from 2,774 Affymetrix U133 Plus 2.0 GeneChips. These were generated from 29 different tumor types which were processed and hybridized at multiple locations worldwide. Data were downloaded from the NCBI GEO repository and processed using the MAS5 algorithm. Normalized genomic profiles were median centered using the 100 internal housekeeping genes present on each chip. The median profile (as defined by expression of the 100 housekeeping genes) in the multi-tumor Expression Project for Oncology (ExpO) dataset was used as the reference profile for this process.

During data pre-processing and normalization, a number of Bioconductor R packages and custom R scripts are employed to assess nine quality variables or metrics on each GeneChip. The complete set of quality data generated during the process is stored in a Microsoft SQL Server 2008 relational database and the values are compared in real-time to predetermined thresholds. All data and results are linked to the users ChipDX account and date/time stamped to allow for future longitudinal and between-treatment-center quality studies.

Tumor samples used to develop the ChipDX Quality Module

Gene expression profiles from the following tumor types were compiled and analyzed in the ChipDX analysis system. Data were obtained in CEL file format. Tumors belonging to classes of less than 3 members are classified as 'other'.

Supplementary Table 2: Tumor types used to develop the ChipDX Quality Module

Tumor type	Number	Tumor type	Number
B-cell Lymphoma	13	Myometrium	3
Bladder	18	Other	19
Breast	336	Ovary	462
Cervix	27	Pancreas	14
Colon	307	Prostate	67
Endometrium	136	Rectum	28
Esophagus	7	Skin neoplasm	7
Gastric	436	Small Intestine	8
Head & Neck	8	Soft tissue	128
Kidney	190	Stomach	6
Liver	120	Testis	5

Tumor type	Number	Tumor type	Number
Lung	237	Thyroid	58
Melanoma	130	Vulva	5

Threshold calculations

To determine a range of acceptable values for each quality metric, without undue influence from far outlier values, we used the median value and interquartile range (IQR) of each metric. In this analysis we define outliers as values more than 1.5 times the IQR above or below the 75th or 25th percentile respectively. Far outliers are those more than 3 times the IQR above or below the 75th or 25th percentiles respectively. Thresholds were then set using the scheme shown in Figure 3.

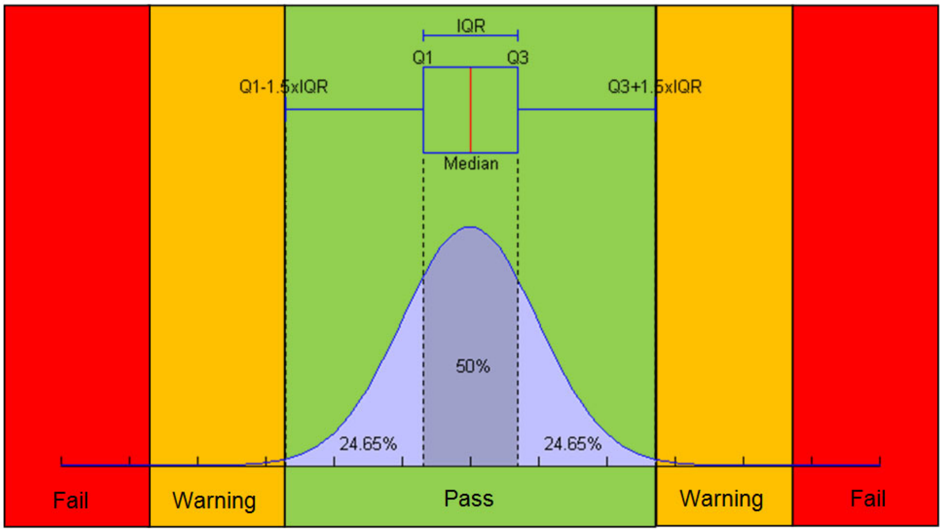

Supplementary Figure 3: Diagram of quality metric thresholds. The nine quality metrics used by the module each approximate a normal distribution after log transformation (where necessary). The thresholds are designed to identify individual measurements that are far outliers, i.e. more than 3*IQR above or below the median value.

Evaluation on real-world tumor datasets

1,710 Affymetrix U133 Plus 2.0 GeneChip hybridizations of RNA from 10 types of primary tumor were analyzed by the Quality Module as part of the ChipDX Tumor Origin Module validation study. Of these,

1,665 (97%) passed all QC metrics and were used for further analysis. An additional set of 224 metastatic tumors were also analyzed, 94% of which satisfied all metrics.

These tumors were downloaded from NCBI GEO and are associated with peer-reviewed publications. As such it is important to note that any very poor quality hybridizations from each original series were most likely excluded as part of the quality control process employed in the respective study. Therefore the 'real-world' percentage of GeneChip hybridizations which pass all 9 metrics used by the Quality Module is likely to be lower.

In order to evaluate whether any tissue-type or location bias (grouping results by Asia-pacific, USA or Europe) existed in the samples excluded, a Kruskal Wallis test was performed. No significant association was observed between tissue type (n=10) or geographic origin and proportion of samples satisfying all components of the quality system (P=0.329 and P=0.429 respectively).

163 probe set and Cox regression p-values.

Supplementary Table 1: Affymetrix Probe IDs and gene annotation for the 163 probe prognostic signature. Cox-regression P-values for each gene are shown, in addition to the relationship between expression and poor prognosis (i.e. high risk).

Affymetrix Probe ID	Name	GB acc	UGCluster	Symbol	P-value (Cox regression, DFS)	Association between expression and poor prognosis
1553954_at	asparagine-linked glycosylation 14 homolog (S. cerevisiae)	BU682208	Hs.408927	ALG14	1.89E-03	-
1554078_s_at	DnaJ (Hsp40) homolog, subfamily A, member 3	BC032100	Hs.719223	DNAJA3	8.51E-04	-
1555832_s_at	Kruppel-like factor 6	BU683415	Hs.4055	KLF6	5.44E-04	-
1555950_a_at	CD55 molecule, decay accelerating factor for complement (Cromer blood group)	CA448665	Hs.126517	CD55	2.32E-05	+
1560089_at	hypothetical LOC286208	AL833509	Hs.693796	LOC286208	1.72E-03	-
1560587_s_at	peroxiredoxin 5	AI718223	Hs.502823	PRDX5	8.98E-04	-
1563796_s_at	glutamyl-tRNA synthetase 2, mitochondrial (putative)	AK095998	Hs.620541	EARS2	1.51E-04	-
200006_at	Parkinson disease (autosomal recessive, early onset) 7	NM_007262	Hs.419640	PARK7	1.88E-03	-
200632_s_at	N-myc downstream regulated 1	NM_006096	Hs.372914	NDRG1	4.74E-05	+
200665_s_at	secreted protein, acidic, cysteine-rich (osteonectin)	NM_003118	Hs.111779	SPARC	9.49E-04	+
200827_at	procollagen-lysine 1, 2-oxoglutarate 5-dioxygenase 1	NM_000302	Hs.75093	PLOD1	1.79E-04	+
200838_at	cathepsin B	NM_001908	Hs.520898	CTSB	1.77E-03	+
200839_s_at	cathepsin B	NM_001908	Hs.520898	CTSB	1.95E-03	+

Affymetrix Probe ID	Name	GB acc	UGCluster	Symbol	P-value (Cox regression, DFS)	Association between expression and poor prognosis
200931_s_at	vinculin	NM_014000	Hs.643896	VCL	5.40E-04	-
200983_x_at	CD59 molecule, complement regulatory protein	BF983379	Hs.278573	CD59	1.20E-03	-
201012_at	annexin A1	NM_000700	Hs.494173	ANXA1	2.47E-04	+
201141_at	glycoprotein (transmembrane) nmb	NM_002510	Hs.190495	GPNMB	1.82E-03	+
201170_s_at	basic helix-loop-helix family, member e40	NM_003670	Hs.719093	BHLHE40	5.20E-06	+
201185_at	HtrA serine peptidase 1	NM_002775	Hs.501280	HTRA1	5.72E-04	+
201261_x_at	biglycan	BC002416	Hs.821	BGN	1.47E-04	+
201289_at	cysteine-rich, angiogenic inducer, 61	NM_001554	Hs.8867	CYR61	7.00E-04	+
201323_at	EBNA1 binding protein 2	NM_006824	Hs.346868	EBNA1BP2	1.65E-03	-
201422_at	interferon, gamma-inducible protein 30	NM_006332	Hs.14623	IFI30	6.79E-04	+
201426_s_at	vimentin	AI922599	Hs.628678	VIM	1.67E-03	+
201578_at	podocalyxin-like	NM_005397	Hs.690098	PODXL	1.27E-03	+
201590_x_at	annexin A2	NM_004039	Hs.511605	ANXA2	5.77E-04	-
201666_at	TIMP metallopeptidase inhibitor 1	NM_003254	Hs.522632	TIMP1	3.55E-04	+
201925_s_at	CD55 molecule, decay accelerating factor for complement (Cromer blood group)	NM_000574	Hs.126517	CD55	2.78E-05	+
201926_s_at	CD55 molecule, decay accelerating factor for complement (Cromer blood group)	BC001288	Hs.126517	CD55	2.68E-05	+
201939_at	polo-like kinase 2 (Drosophila)	NM_006622	Hs.398157	PLK2	1.45E-03	+
201951_at	activated leukocyte cell adhesion molecule	BF242905	Hs.591293	ALCAM	2.13E-04	+
202068_s_at	low density lipoprotein receptor	NM_000527	Hs.213289	LDLR	1.02E-04	+
202237_at	nicotinamide N-methyltransferase	NM_006169	Hs.503911	NNMT	1.80E-03	+
202238_s_at	nicotinamide N-methyltransferase	NM_006169	Hs.503911	NNMT	1.80E-03	+
202419_at	3-ketodihydrosphingosine reductase	NM_002035	Hs.74050	KDSR	4.95E-04	+
202457_s_at	protein phosphatase 3 (formerly 2B), catalytic subunit, alpha isoform	AA911231	Hs.435512	PPP3CA	1.90E-03	+
202478_at	tribbles homolog 2 (Drosophila)	NM_021643	Hs.467751	TRIB2	7.90E-04	+
202839_s_at	NADH dehydrogenase (ubiquinone) 1 beta subcomplex, 7, 18kDa	NM_004146	Hs.532853	NDUFB7	6.09E-04	-
202887_s_at	DNA-damage-inducible transcript 4	NM_019058	Hs.523012	DDIT4	8.94E-05	+
202904_s_at	LSM5 homolog, U6 small nuclear RNA associated (S. cerevisiae)	NM_012322	Hs.424908	LSM5	1.97E-03	-
202939_at	zinc metallopeptidase (STE24 homolog, S. cerevisiae)	NM_005857	Hs.132642	ZMPSTE24	1.79E-03	-
202949_s_at	four and a half LIM domains 2	NM_001450	Hs.443687	FHL2	2.82E-04	-
203072_at	myosin IE	NM_004998	Hs.654506	MYO1E	8.77E-04	+
203083_at	thrombospondin 2	NM_003247	Hs.371147	THBS2	1.23E-04	+
203382_s_at	apolipoprotein E	NM_000041	Hs.654439	APOE	4.30E-04	+
203476_at	trophoblast glycoprotein	NM_006670	Hs.82128	TPBG	1.50E-04	+
203895_at	phospholipase C, beta 4	AL535113	Hs.472101	PLCB4	6.44E-04	-
204264_at	carnitine palmitoyltransferase 2	NM_000098	Hs.713535	CPT2	9.97E-04	-
204472_at	GTP binding protein overexpressed in skeletal muscle	NM_005261	Hs.654463	GEM	4.33E-04	+
204620_s_at	versican	NM_004385	Hs.643801	VCAN	5.28E-04	+
204679_at	potassium channel, subfamily K, member 1	NM_002245	Hs.208544	KCNK1	1.58E-03	+
205677_s_at	deleted in lymphocytic leukemia 1 (non-protein coding)	NM_005887	Hs.591229	DLEU1	7.15E-04	-
205963_s_at	DnaJ (Hsp40) homolog, subfamily A, member 3	NM_005147	Hs.719223	DNAJA3	4.48E-04	-
207543_s_at	prolyl 4-hydroxylase, alpha polypeptide I	NM_000917	Hs.500047	P4HA1	1.62E-05	+

Affymetrix Probe ID	Name	GB acc	UGCluster	Symbol	P-value (Cox regression, DFS)	Association between expression and poor prognosis
207574_s_at	growth arrest and DNA-damage-inducible, beta	NM_015675	Hs.110571	GADD45B	4.19E-04	+
208891_at	dual specificity phosphatase 6	BC003143	Hs.298654	DUSP6	5.66E-04	+
208892_s_at	dual specificity phosphatase 6	BC003143	Hs.298654	DUSP6	1.70E-03	+
208893_s_at	dual specificity phosphatase 6	BC005047	Hs.298654	DUSP6	1.45E-03	+
208918_s_at	NAD kinase	AI334128	Hs.654792	NADK	7.87E-04	-
208961_s_at	Kruppel-like factor 6	AB017493	Hs.4055	KLF6	1.75E-03	+
209043_at	3'-phosphoadenosine 5'-phosphosulfate synthase 1	AF033026	Hs.368610	PAPSS1	4.70E-04	-
209101_at	connective tissue growth factor	M92934	Hs.591346	CTGF	8.53E-05	+
209184_s_at	insulin receptor substrate 2	BF700086	Hs.442344	IRS2	8.39E-04	-
209185_s_at	insulin receptor substrate 2	AF073310	Hs.442344	IRS2	5.24E-04	-
209193_at	pim-1 oncogene	M24779	Hs.81170	PIM1	7.01E-04	+
209345_s_at	phosphatidylinositol 4-kinase type 2 alpha	AL561930	Hs.25300	PI4K2A	1.53E-03	+
209386_at	transmembrane 4 L six family member 1	AI346835	Hs.715499	TM4SF1	2.74E-05	-
209387_s_at	transmembrane 4 L six family member 1	M90657	Hs.715499	TM4SF1	1.10E-03	+
209457_at	dual specificity phosphatase 5	U16996	Hs.2128	DUSP5	1.71E-03	+
209545_s_at	receptor-interacting serine-threonine kinase 2	AF064824	Hs.103755	RIPK2	1.57E-03	+
209624_s_at	methylcrotonoyl-Coenzyme A carboxylase 2 (beta)	AB050049	Hs.604789	MCCC2	1.21E-03	-
209711_at	solute carrier family 35 (UDP-glucuronic acid/UDP-N-acetylgalactosamine dual transporter), member D1	N80922	Hs.213642	SLC35D1	1.70E-04	-
209875_s_at	secreted phosphoprotein 1	M83248	Hs.313	SPP1	1.88E-04	+
210095_s_at	insulin-like growth factor binding protein 3	M31159	Hs.450230	IGFBP3	6.96E-04	+
210275_s_at	zinc finger, AN1-type domain 5	AF062347	Hs.406096	ZFAND5	6.18E-04	-
210427_x_at	annexin A2	BC001388	Hs.511605	ANXA2	1.57E-03	-
210495_x_at	fibronectin 1	AF130095	Hs.203717	FN1	4.08E-05	+
210512_s_at	vascular endothelial growth factor A	AF022375	Hs.73793	VEGFA	3.54E-05	-
210517_s_at	A kinase (PRKA) anchor protein 12	AB003476	Hs.371240	AKAP12	1.99E-04	+
210592_s_at	spermidine/spermine N1-acetyltransferase 1	M55580	Hs.28491	SAT1	7.13E-04	-
210652_s_at	tetratricopeptide repeat domain 39A	BC004399	Hs.112949	TTC39A	1.64E-03	-
210845_s_at	plasminogen activator, urokinase receptor	U08839	Hs.466871	PLAUR	1.20E-04	+
211074_at	folate receptor 1 (adult)	AF000381	Hs.73769	FOLR1	1.81E-05	-
211719_x_at	fibronectin 1	BC005858	Hs.203717	FN1	1.91E-04	+
211924_s_at	plasminogen activator, urokinase receptor	AY029180	Hs.466871	PLAUR	1.10E-03	+
211928_at	dynein, cytoplasmic 1, heavy chain 1	AB002323	Hs.649497	DYNC1H1	1.01E-03	+
211988_at	SWI/SNF related, matrix associated, actin dependent regulator of chromatin, subfamily e, member 1	BG289800	Hs.696086	SMARCE1	1.51E-03	-
212013_at	peroxidasin homolog (Drosophila)	D86983	Hs.332197	PXDN	2.74E-04	+
212143_s_at	insulin-like growth factor binding protein 3	BF340228	Hs.450230	IGFBP3	1.82E-03	+
212171_x_at	vascular endothelial growth factor A	H95344	Hs.73793	VEGFA	8.33E-04	-
212463_at	CD59 molecule, complement regulatory protein	BE379006	Hs.278573	CD59	1.02E-03	-
212464_s_at	fibronectin 1	X02761	Hs.203717	FN1	3.36E-05	+
212501_at	CCAAT/enhancer binding protein (C/EBP), beta	AL564683	Hs.517106	CEBPB	8.65E-04	-
212632_at	syntaxin 7	N32035	Hs.593148	STX7	8.03E-04	+
212884_x_at	apolipoprotein E	AI358867	Hs.654439	APOE	2.19E-04	+

Affymetrix Probe ID	Name	GB acc	UGCluster	Symbol	P-value (Cox regression, DFS)	Association between expression and poor prognosis
213274_s_at	cathepsin B	AA020826	Hs.520898	CTSB	1.77E-03	+
213503_x_at	annexin A2	BE908217	Hs.511605	ANXA2	7.82E-04	+
213905_x_at	biglycan	AA845258	Hs.821	BGN	2.69E-04	+
214581_x_at	tumor necrosis factor receptor superfamily, member 21	BE568134	Hs.443577	TNFRSF21	1.24E-03	+
214620_x_at	peptidylglycine alpha-amidating monooxygenase	BF038548	Hs.369430	PAM	6.78E-04	+
214866_at	plasminogen activator, urokinase receptor	X74039	Hs.466871	PLAUR	4.11E-04	+
215033_at	transmembrane 4 L six family member 1	AI189753	Hs.715499	TM4SF1	2.05E-05	-
215034_s_at	transmembrane 4 L six family member 1	AI189753	Hs.715499	TM4SF1	2.05E-05	+
215792_s_at	DnaJ (Hsp40) homolog, subfamily C, member 11	AL109978	Hs.462640	DNAJC11	1.81E-03	-
216392_s_at	SEC23 interacting protein	AK021846	Hs.435004	SEC23IP	5.52E-04	+
216442_x_at	fibronectin 1	AK026737	Hs.203717	FN1	2.37E-05	+
217762_s_at	RAB31, member RAS oncogene family	BE789881	Hs.714730	RAB31	1.32E-03	+
217773_s_at	NADH dehydrogenase (ubiquinone) 1 alpha subcomplex, 4, 9kDa	NM_002489	Hs.50098	NDUFA4	1.86E-05	-
217996_at	pleckstrin homology-like domain, family A, member 1	AA576961	Hs.602085	PHLDA1	4.74E-04	+
218213_s_at	chromosome 11 open reading frame 10	NM_014206	Hs.437779	C11orf10	1.63E-03	-
218698_at	APAF1 interacting protein	NM_015957	Hs.447794	APIP	1.77E-03	-
218856_at	tumor necrosis factor receptor superfamily, member 21	NM_016629	Hs.443577	TNFRSF21	8.15E-04	+
218902_at	Notch homolog 1, translocation-associated (Drosophila)	NM_017617	Hs.495473	NOTCH1	5.32E-04	-
219038_at	MORC family CW-type zinc finger 4	NM_024657	Hs.496544	MORC4	6.74E-04	+
219206_x_at	transmembrane BAX inhibitor motif containing 4	NM_016056	Hs.505934	TMBIM4	1.51E-03	-
219539_at	gem (nuclear organelle) associated protein 6	NM_024775	Hs.143818	GEMIN6	1.92E-03	-
221419_s_at		NM_013307			5.04E-04	-
221479_s_at	BCL2/adenovirus E1B 19kDa interacting protein 3-like	AF060922	Hs.131226	BNIP3L	2.06E-04	+
221563_at	dual specificity phosphatase 10	N36770	Hs.497822	DUSP10	7.92E-04	+
221648_s_at		AK025651			1.07E-03	-
221656_s_at	Rho guanine nucleotide exchange factor (GEF) 10-like	BC003073	Hs.443460	ARHGEF10L	1.20E-03	-
221730_at	collagen, type V, alpha 2	NM_000393	Hs.445827	COL5A2	1.86E-03	+
221731_x_at	versican	BF218922	Hs.643801	VCAN	1.88E-03	+
221745_at	DDB1 and CUL4 associated factor 7	BE538424	Hs.410596	DCAF7	1.75E-03	-
222421_at	ubiquitin-conjugating enzyme E2H (UBC8 homolog, yeast)	BF435617	Hs.643548	UBE2H	1.66E-03	-
222994_at	peroxiredoxin 5	AF197952	Hs.502823	PRDX5	1.02E-03	-
223003_at	chromosome 19 open reading frame 43	AF061732	Hs.515155	C19orf43	1.67E-03	-
223122_s_at	secreted frizzled-related protein 2	AF311912	Hs.481022	SFRP2	3.15E-05	+
223163_s_at	zinc finger, C3HC-type containing 1	BC000190	Hs.194157	ZC3HC1	1.94E-03	-
223312_at	chromosome 2 open reading frame 7	BC005069	Hs.61268	C2orf7	4.95E-05	-
223454_at	chemokine (C-X-C motif) ligand 16	AF275260	Hs.711652	CXCL16	8.98E-04	+
223455_at	trichoplein, keratin filament binding	BG493862	Hs.410924	TCHP	3.80E-04	-
224602_at	chromosome 4 open reading frame 3	BF244081	Hs.701808	C4orf3	1.61E-03	+
224606_at	Kruppel-like factor 6	BG250721	Hs.4055	KLF6	1.91E-04	-
224657_at	ERBB receptor feedback inhibitor 1	AL034417	Hs.605445	ERRFI1	1.29E-03	+

Affymetrix Probe ID	Name	GB acc	UGCluster	Symbol	P-value (Cox regression, DFS)	Association between expression and poor prognosis
224777_s_at	platelet-activating factor acetylhydrolase, isoform Ib, subunit 2 (30kDa)	BG386322	Hs.718419	PAFAH1B2	1.81E-03	-
224806_at	tripartite motif-containing 25	BE563152	Hs.528952	TRIM25	1.54E-04	-
224890_s_at	chromosome 7 open reading frame 59	BE727643	Hs.406520	C7orf59	1.32E-03	-
224911_s_at	discoidin, CUB and LCCL domain containing 2	AA722799	Hs.203691	DCBLD2	1.74E-03	+
225010_at	coiled-coil domain containing 6	AK024913	Hs.591360	CCDC6	1.49E-03	-
225011_at	protein kinase, cAMP-dependent, regulatory, type II, alpha	AK026351	Hs.631923	PRKAR2A	4.84E-04	-
225337_at	abhydrolase domain containing 2	AI346910	Hs.122337	ABHD2	1.55E-03	+
225494_at	dynein, light chain, LC8-type 2	BG478726	Hs.514368	DYNLL2	1.17E-04	-
225670_at	family with sequence similarity 173, member B	AI384017	Hs.481569	FAM173B	8.18E-04	-
225750_at		BE966748			6.24E-04	-
226041_at	N-acyl phosphatidylethanolamine phospholipase D	BF382393	Hs.324271	NAPEPLD	1.87E-03	-
226594_at		AA528157	Hs.655070		1.12E-03	-
226648_at	hypoxia inducible factor 1, alpha subunit inhibitor	AI769745	Hs.500788	HIF1AN	1.93E-03	-
226727_at	CDGSH iron sulfur domain 3	BG171264	Hs.713595	CISD3	3.53E-04	-
226987_at	RNA binding motif protein 15B	W68720	Hs.118738	RBM15B	1.48E-03	-
227143_s_at	BH3 interacting domain death agonist	AA706658	Hs.591054	BID	1.30E-03	-
227338_at	hypothetical gene supported by BC066916	H99038	Hs.535002	LOC440983	7.99E-04	-
227735_s_at		AA553959	Hs.298713	C10orf99	9.29E-04	-
227736_at	chromosome 10 open reading frame 99	AA553959	Hs.298713	C10orf99	2.00E-03	-
227961_at	cathepsin B	AA130998	Hs.520898	CTSB	1.94E-03	+
229676_at	mitochondrial poly(A) polymerase	AA400998	Hs.173946	MTPAP	2.41E-05	-
231576_at		AA829940	Hs.713786		9.56E-05	-
234983_at		BE893995	Hs.120170		1.10E-04	-
241355_at	hairless homolog (mouse)	BF528433	Hs.272367	HR	1.20E-03	-
242648_at	kelch-like 8 (Drosophila)	BE858995	Hs.106601	KLHL8	1.59E-03	-
35156_at	R3H domain and coiled-coil containing 1	AL050297	Hs.458644	R3HCC1	1.37E-03	-
36711_at	v-maf musculoaponeurotic fibrosarcoma oncogene homolog F (avian)	AL021977	Hs.517617	MAFF	1.77E-03	+
58780_s_at	hypothetical protein FLJ10357	R42449	Hs.35125	FLJ10357	7.64E-04	-
